# Editorial: Bacteriocin-Producing Probiotic Bacteria: A Natural Solution for Increasing Efficiency and Safety of Livestock Food Production

**DOI:** 10.3389/fmicb.2021.675483

**Published:** 2021-05-28

**Authors:** Ramona Iseppi, Andrea Lauková, Carla Sabia

**Affiliations:** ^1^Department of Life Sciences, University of Modena and Reggio Emilia Modena, Modena, Italy; ^2^Centre of Biosciences of the Slovak Academy of Sciences, Institute of Animal Physiology, Košice, Slovakia

**Keywords:** probiotic, bacteriocin, lactobacilli, livestock food production, safety

In modern animal husbandry, animal feed represents one of the key points to reach competitive production level, both qualitatively and quantitatively. Nowadays, in addition to the numerous raw materials on the market, we have access to a lot of modern technologies that allow to obtain more better formulated products with optimal characteristic of palatability, digestibility, shelf-life, and stability. Also, in animal nutrition substances called food additives are even more frequently employed. They are intended to improve the technical and qualitative characteristics of the food in which they are inserted, and to optimize the metabolic-digestive processes of animals, e.g., in use of probiotic bacteria.

For this reason and also for harmonizing the consumer demands to reach the requested safety standard, the research has been focused on a natural preservation approach. Natural substances with an antimicrobial activity have already been identified from a large variety of sources such as herbs, medicinal plants, microorganisms, and animals (Naimi et al.). Among them lactic acid bacteria (LABs) generally recognized as safe (GRAS) by the Food and Drug Administration (FDA), are most frequently applied in different kind of human and animal food with an effective activity to control or counteract pathogenic (including those antibiotic-resistant) and spoilage bacteria (Akter et al.). LABs are present in food, environment as well as in human and animal gut, although fermented foods (FFs) are recognized as the primary niche of LABs activity. LAB exhibit considerable species and strain diversity and can play a significant role in different ecosystems, nevertheless food remains their major source and preferred activity niche. Many foods are enriched with probiotic LAB to reset intestinal microbiota or maintain it well-balanced. Probiotics have been defined as a live microbial feed supplements, which beneficially affect animal or human host by improving the intestinal balance (Kosin and Rakshit, 2006). Some strains of LAB are considered as alternative to bio-conservatives in food and breeding because they can produce molecules with anti-bacterial activity such as lactic acid, hydrogen peroxide, and in particular they can produce bacteriocins. Bacteriocins are antimicrobial proteins/peptides produced by Gram-negative and mostly by Gram-positive bacteria, including LAB. LAB are also those organisms that are traditionally used in food preservation practices and/or in feed industry as natural preservatives and probiotics as well (Diez-Gonzalez, 2007; Naimi et al.).

In a recent study, a LAB strain OSY-TC318, was isolated from a Turkish cheese. The crude extract of the cultured strain inhibited the growth of various pathogenic and spoilage bacteria such as *Bacillus cereus, Clostridium sporogenes, Enterococcus faecalis, Listeria monocytogenes, Salmonella enterica ser. Typhimurium*, and *Staphylococcus aureus* (Hussein, Huang et al.). A genome-guided mass spectrometric analysis was used to rapidly reveal the structure of paraplantaricin TC318, produced by *Lactobacillus paraplantarum* TC318 and it is a novel member of class I lantibiotics. The bacteriocin paraplantaricin TC318 is potentially useful in food or in medical application. At second study, an antimicrobial peptide (AMP) of the mass 5 kDa, produced by *Lactobacillus acidophilus* ATCC-4356/DSM20079, with inhibition activity against *Aeromonas hydrophila* (Akter et al.) was presented. *L. acidophilus* may be suitable candidates for the long-term control of *A. hydrophila* infection and can be introduced into fish feed, which is the best way to administer “drug” candidate. The antimicrobial peptide (AMP) produced by probiotic strain may be possible “drug” candidate for *A. hydrophila* infection in aquaculture. The third research showed the safety of *Enterococcus durans* OSY-EGY as a probiotic retrieved from an fermented Turkish cheeses. The *in silico* comparative genomic analysis determined *E. durans* OSY-EGY distance from potentially pathogenic strains as well as the lack of antibiotic resistance and virulence factor genes. The antibiotic susceptibility test revealed that *E. durans* OSY-EGY was susceptible to clinically relevant antibiotics including ampicillin, vancomycin, tetracycline, and aminoglycosides. According to EFSA guidelines, it was considered safe and usable as feed additive. The bioinformatic analysis revealed genes associated with desirable probiotic traits such as acid tolerance, bile tolerance, competitiveness, persistence, adherence, and health promoting effect (Hussein, Abdelhamid et al.). This supports the notion that the strain is potentially suitable for use in various applications. As formerly mentioned, bacteriocins can be also produced by Gram-negative bacteria. In recent research, a 21-amino acid bacteriocin produced by *Escherichia coli* (Microcin J25) was found to have potential antibacterial activity against *Salmonella enterica* subsp. *enterica* serovar Newport ATCC 6962 (*Salmonella* Newport) (Naim et al.). The inhibition activity of MccJ25 against *Salmonella* Newport was examined in Luria-Bertan broth (LB) and in modified MacFarlane medium to mimic the swine colonic condition. The MccJ25 activity was further determined using the Polyfermentor intestinal model (PolyFermS), an *in vitro* continuous fermentation model that allows to decipher the activity of any antimicrobial molecule in real colon fermentation condition using selected microbiota. This bacteriocin might be considered a good alternative to antibiotics for animal feed application; although further *ex vivo* and *in vivo* studies should be focused on validating the effectiveness of MccJ25 as an inhibitor against *Salmonella* spp. in farmed pigs as well as evaluating its impact on the intestinal microbiota.

The results of these research studies show that the antibacterial peptides tested, produced by probiotic bacteria, indicate a viable alternative to reduce the proliferation of pathogenic bacteria, the problem of antibiotic resistance and toxin-producing bacteria in food and feed.

## Author Contributions

RI, CS, and AL: conceptualization and validation. CS: supervision. All authors have read and agreed to the published version of the manuscript.

## Conflict of Interest

The authors declare that the research was conducted in the absence of any commercial or financial relationships that could be construed as a potential conflict of interest.
